# Three-Dimensional Imaging by Frequency-Comb Spectral Interferometry

**DOI:** 10.3390/s20061743

**Published:** 2020-03-20

**Authors:** Haihan Zhao, Ziqiang Zhang, Xinyang Xu, Haoyun Zhang, Jingsheng Zhai, Hanzhong Wu

**Affiliations:** 1 School of Marine Science and Technology, Tianjin University, Tianjin 300072, China; zhaohaihan@tju.edu.cn (H.Z.); xuxinyang@tju.edu.cn (X.X.); zhanghaoyun@tju.edu.cn (H.Z.); jingsheng@tju.edu.cn (J.Z.); 2 Beijing Aerospace Xinfeng Mechanical Equipment Co., LTD, Beijing 100854, China; htjc283@outlook.com

**Keywords:** frequency comb, spectral interferometry, mode-locked laser, 3D imaging

## Abstract

In this paper, we demonstrate a three-dimensional imaging system based on the laser frequency comb. We develop a compact, all-fiber mode-locked laser at 1 μm, whose repetition frequency can be tightly synchronized to the external frequency reference. The mode-locked state is achieved via the saturable absorber mirror in a linear cavity, and the laser output power can be amplified from 4 mW to 150 mW after a Yb-doped fiber amplifier. Three-dimensional imaging is realized via the spectral interferometry with the aid of an equal-arm Michelson interferometer. Compared with the reference values, the measurement results show the difference can be below 4 μm. Our system could provide a pathway to the real industry applications in future.

## 1. Introduction

Frequency combs, which are composed of a series of evenly-spaced single lines across a broad spectral band and correspond to an ultrashort pulse train in the time domain, have seen a vast number of applications in both science and technology [1]. There are two key parameters—the repetition frequency and the carrier-envelope-offset frequency—to precisely characterize the individual frequency markers. In general, the repetition frequency can be measured and stabilized easily, while the detection and lock of the carrier-envelope-offset frequency is challenging [2,3]. Since the first stabilization of carrier envelope phase (CEP) with f-to-2f self-referencing scheme, a wealth of applications have been significantly triggered with great progress, e.g., optical frequency metrology [4], precision spectroscopy [5], optical clock [6], optical communication [7], and absolute distance measurement [8], etc. The imaging technique is of crucial importance in the fields ranging from natural science to the real industry. Generally, the photogrammetry can capture the measured specimens in a large field of view, with appropriate process of the photographs. To realize the three-dimensional imaging, two or more cameras are required [9]. This makes the system complicated, and the measurement precision is only tens of micrometers. The coordinate measuring machine CMM can be used to image the target surface with high precision (several micrometers), based on the point cloud after a continuous scan of the specimen [10]. However, the probe is needed to travel on the surface, which could scratch the specimen. The CMM itself is bulky and expensive. Additionally, the ultrasonic based method can be also used to image the specimen roughly [11]. In contrast, optical imaging methods preserve the advantages of, e.g., high precision, cost efficiency, non-contact and non-intrusive measurements [12], which is attractive to real industrial applications.

Frequency combs can serve as the laser source in the three-dimensional imaging, indirectly and directly. In the former case and typically, a continuous wave laser, working as the measuring source, is stabilized by the frequency comb [13,14]. The step height of the gauge blocks is measured via the relative phase change of the continuous wave laser. Due to the stabilized wavelength of the continuous laser, the measurement uncertainty can reach about 15 nm. Recently, a frequency modulated continuous wave LiDAR enabling three-dimensional imaging at a distance was demonstrated [15,16], where the external cavity diode laser is calibrated by a mode-locked laser. The sweeping nonlinearity can be measured and compensated in real-time with the help of the mode-locked laser, and the measurement precision can achieve 10 μm. However, multi laser sources are required, and therefore the resulting system is large-size, complicated, environment-sensitive, and expensive. Directly, dual-comb three-dimensional imaging has been reported, by using the electro-optic frequency combs [17,18], and Kerr soliton combs [19], respectively. All these methods rely upon the phase measurement of the multi heterodyne interferometry. However, two well–synchronized comb lasers are required. For these three kinds of combs, two phase-stable combs are expensive, and great effort is needed to build the entire measurement system reliably. The schemes utilizing the single comb source are also described, e.g., optical sampling by cavity tuning [20], and chirped pulse interferometry [21]. The method of optical sampling by cavity tuning can be treated as the simplified dual-comb sampling, where the optical sampling is realized by the sweeping of the repetition frequency with a long fiber delay link. When the interferograms are obtained, the three-dimensional surface can be reconstructed. Analogously, in the case of chirped pulse interferometry, one can use the position of the widest fringe in the spectral interferograms to determine the step height of the specimen [22]. However, the entire system is still often bulky and vibration-sensitive, which strongly hinders the real field-base applications of frequency combs. In past periods, we visited many factories of precision manufacturing, and found that the technique of three-dimensional imaging is urgently needed on occasions such as the shape/dimensional (including the weld and solder joint) measurement of the workpiece, and the screw height measurement, etc. Frequency comb based three-dimensional imaging can be an outstanding solution. To meet the requirements of the field/industry applications, the first challenge is to develop a portable, and environment-insensitive frequency comb laser source, and the second goes to a powerful method to measure the three-dimensional profile with high precision.

In this Letter, we demonstrate a three-dimensional imager based on the frequency-comb spectral interferometry, and only one comb laser was needed. We first developed a small-size, all-fiber mode-locked laser at 1 μm. The passive mode-locked state is achieved by using a saturable absorber mirror. After the Yb–doped fiber power amplifier, the laser output can be used to irradiate the specimen fixed on a two-axis platform. The three–dimensional imaging of the specimen can be realized based on the point cloud obtained by the spectral interferometry.

## 2. Methods

**Figure 1:**
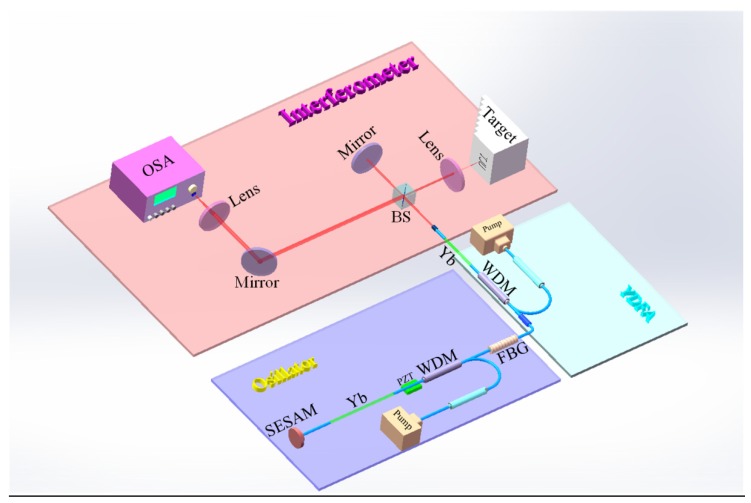
Experimental schematic of the three-dimensional imager, which is composed of three parts: oscillator (purple background), YDFA (light blue background), and interferometer (light red background). SESAM: semiconductor saturable absorber; PZT: piezoelectric transducer; WDM: wavelength division multiplexing; FBG: fiber Bragg grating; YDFA: Yb–doped fiber amplifier; BS: beam splitter; OSA: optical spectrum analyzer.

The experimental setup is shown in Figure 1, which consists of three parts in fact: the oscillator (mode-locked laser), the power amplifier, and the Michelson interferometer. In the mode-locked laser, the pump laser diode is II–VI LC96Z600–76 (976 nm, 600 mW maximum output power), which is injected into the cavity after a fiber isolator. The linear cavity is composed of a fiber Bragg grating FBG (0.8 nm spectral width, 87% reflectance), a wavelength division multiplexing WDM, Yb–doped gain fiber (Liekki, Yb1200–4/125, 13.5 cm), and a saturable absorber mirror SAM (BATOP, SAM-1030-32-1ps). FBG and SAM actually work as the two cavity boundaries. When the pump power reaches about 40 mW, the mode-locked state can be achieved, and the laser source can stably output a pulse train, with 4 mW output power and 28.5 MHz repetition frequency. A Yb-doped fiber amplifier YDFA is designed to amplify the laser power to about 150 mW.

**Figure 2:**
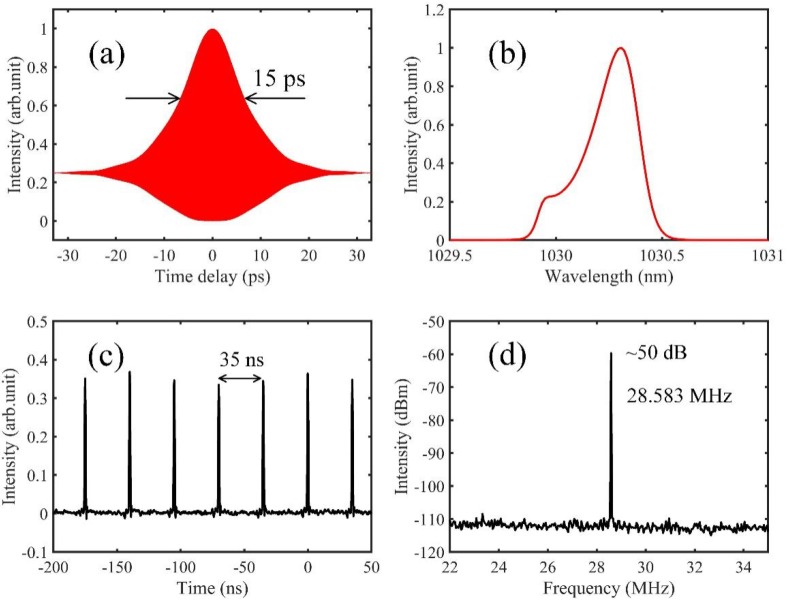
Characterization of the frequency comb source. (**a**) Pulse width measured by an autocorrelator. The pulse width is found as 15 ps; (**b**): Optical spectrum of the frequency comb source, measured by an optical spectrum analyzer (Thorlabs OSA201C); (**c**) Intracavity waveform, which is a stable pulse train with about 35 ns period; (**d**) Electrical spectrum, measured by a spectrum analyzer (Rigol DSA832) with 300 Hz RBW. The repetition frequency is 28.583 MHz with 50 dB signal-to-noise ratio.

Figure 2a shows the pulse width measured by an autocorrelator, and the pulse width is about 15 ps. Figure 2b indicates the spectrum, which is centered at 1030 nm with about 0.8 nm width (corresponding to the bandwidth of the fiber Bragg grating). Figure 2c shows the intracavity waveform in the time domain, which is a stable pulse train with 35 ns time interval. Figure 2d depicts the electrical spectrum of the output laser, and the repetition frequency is about 28.583 MHz with over 50 dB signal-to-noise ratio. The repetition frequency can be precisely locked to the frequency reference (See Appendix A, for the demonstrations in detail). The output of the amplifier is guided into a Michelson interferometer, which is split to two parts at the beam splitter. One part is reflected by the reference mirror, and the other is focused onto the specimen, and back scattered on the surface of the specimen. The two beams are combined at the beam splitter, and detected by an optical spectrum analyzer. The specimen is fixed on a two-axis platform. Based on the theory of spectral interferometry [23], the spectrum will be modulated by a cosine signal, when the reference and measurement beams interfere with each other in the frequency domain. The modulated spectrum can be simply expressed as:

(1) $$I(\omega )=E^{2}(\omega )\cdot \left[1+\cos(\tau \omega )\right]$$

where *I*(*ω*) is the interfered spectral power, *E*(*ω*) is the source spectrum, *ω* is the optical angular frequency, and *τ* is the time delay between the reference and measurement beams. The optical path difference *d* between the reference and measurement beams can be therefore calculated as:

(2) $$d=\frac{1}{2}\cdot \frac{c}{n_{g}}\cdot \frac{\partial \varphi }{\partial \omega }$$

where *c* is the light speed in vacuum, *n_g_* is the group refractive index corrected by Ciddor formula [24], and *φ* = *τω*, the unwrapped phase of the spectral interferograms. In general, the slope of the unwrapped phase (i.e., *∂φ*/*∂ω*) can be precisely determined via fast Fourier transform [23], and consequently, *d* can be obtained. It is obvious that *d* will change if the specimen is scanned in a two-axis plane (e.g., *x* − *y* plane). The three-dimensional image can be measured as long as we plot the point cloud in a three-axis coordinate system (e.g., *x* − *y* − *d* coordinate system). In our measurement system, the moving range of the *x* and *y* axis is 50 mm. The measurable range of *d* is related to the spectral resolution of the optical spectral analyzer. 

## 3. Results and Discussion

**Figure 3:**
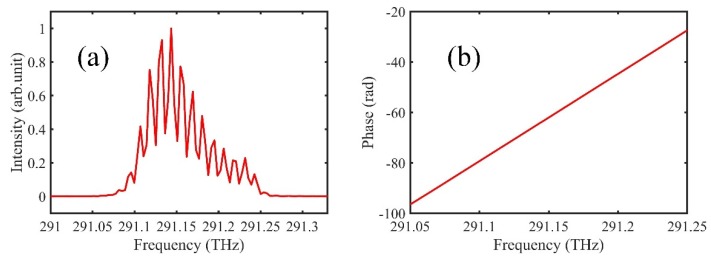
(**a**) Measured spectral interferogram. The x-axis is optical frequency; (**b**) Unwrapped phase obtained by fast Fourier transform. The slope of the unwrapped phase can be easily measured.

We first measure a customized specimen, with a series of step heights and a letter pattern (i.e., tju). The environmental conditions are 24.08 °C, 991.93 hPa, and 43.5% humidity, and the group refractive index is 1.0002624 based on Ciddor formula. The measured spectral interferograms are shown in Figure 3, and the slope of the unwrapped phase can be obtained by fast Fourier transform, which can be used to calculate the instantaneous distances.

**Figure 4:**
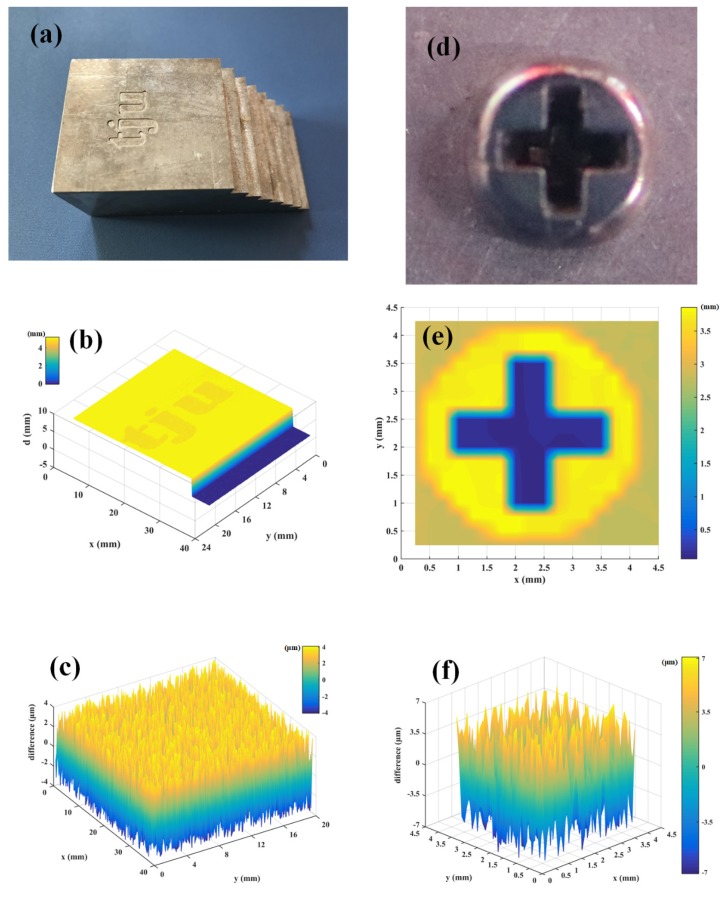
Experimental results of three-dimensional imaging. (**a**) Photograph of the customized specimen; (**b**) Reconstructed image based on the point cloud. We find that the “tju” letters can be clearly resolved; (**c**) Difference between our measurement results and the reference values; (**d**) Photograph of the screw specimen; (**e**) Reconstructed image based on the point cloud. The cross hole can be measured clearly; (**f**) Difference between our measurement results and the reference values, for the screw.

The photograph of the customized specimen is shown in Figure 4a, and the depth of the letters is about 200 μm. The step height is about 5 mm. In our experiments, the step size of the scanning platforms is fixed to 200 μm. Figure 4b shows the point cloud in a three-axis coordinate system. We find that, the letters ‘tju’ can be clearly resolved, and the step height can be also measured. The specimens are also measured by a commercial optical microscope (Bruker ContourGT-K), whose measurement results are used as the reference values. Compared with the reference value, our experimental results show the difference within 4 μm, as shown in Figure 4c. Therefore, our system can realize three-dimensional imaging with high accuracy and precision. To access the real industry applications, we measure a screw specimen, and the results are shown in Figure 4e. Figure 4d indicates a photograph of the screw specimen. We find, the screw can be also well captured, and the difference between our results and the reference values is less than 7 μm, as shown in Figure 4f. The measuring time for one single point is about 150 ms. The point number is 7878 for the ‘tju’ specimen, and 306 for the screw, respectively. Therefore, the total measuring time is 1.182 s for the ‘tju’ specimen, and 46 s for the screw, respectively.

**Figure 5:**
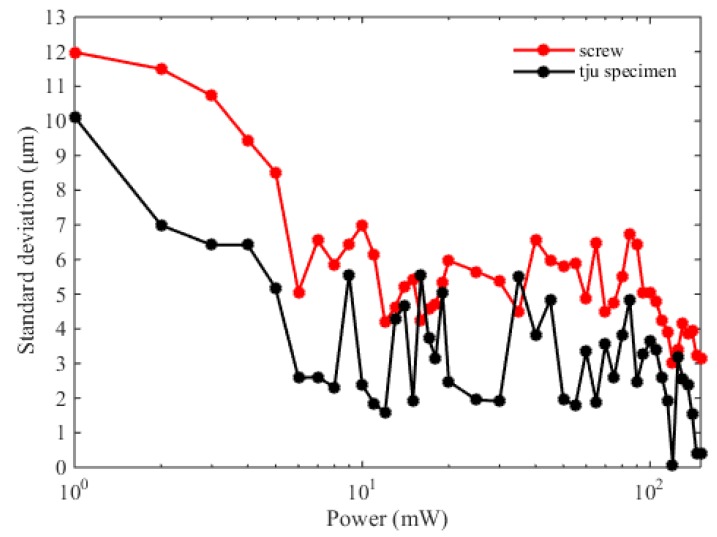
Standard deviations with different irradiation power. The black solid points indicate the standard deviation with five measurements, for the tju specimen, and the red solid points show the standard deviation for the screw.

Based on the results shown in Figure 4, we find that the specimen can be well imaged using the frequency-comb spectral interferometry, which exhibits great potential in the real industry applications. Note that compared with the experiments in the clean lab, one key point is that the target is not a high-reflection mirror, but an arbitrary object. In this case, the object properties, e.g., temperature, material, roughness, color, and reflectivity, etc. will make great contributions to the measurement results. All of these factors can be attributed to the optical power of the back-scattered light. Therefore, we test the measurement performance with different irradiation power from 1 mW to 150 mW, and the standard deviation is used here to evaluate the measurement results. In our experiments, the specimen is fast measured for five times at the same point, with each different power. The experimental results are shown in Figure 5, which clearly indicates that the standard deviation decreases with increasing irradiation power (from 1 mW to 150 mW). The black solid points depict the standard deviation for the “tju” specimen. We have found that the standard deviation can be up to 10.12 μm with 1 mW irradiation power, and less than 3 μm when the output power is larger than 120 mW. The red solid points represent the standard deviation for the screw, which is slightly larger than that for the tju specimen. When the irradiation power is above 120 mW, the standard deviation can be below 5 μm. We consider that this is because the screw in the practical environment could be covered by some pollutions (e.g., oil, dust), which can reduce the power of the backscattered light. Our experimental results show that higher irradiation power is favorable in the real industry applications with targets, not a high-reflection mirror.

In discussion, the measurement uncertainty is related to the air refractive index *n_g_* and the time delay *τ* based on the Equation (2), which can be calculated as:

(3) $$u_{D}=\sqrt{{\left(\frac{D}{n_{g}}\cdot u_{n_{g}}\right)}^{2}+\frac{1}{2}\cdot {\left(\frac{c}{n_{g}}\cdot u_{\tau }\right)}^{2}}$$

where *D* is the measured depth of the letters. We consider the case of the tju specimen first. The first term in equation (3) is related to the air refractive index *n_g_*. In our work, the air refractive index is corrected by the Ciddor formula based on the environmental sensor networks. The inherent uncertainty of the empirical equation is 2 × 10^−8^. Considering the inherent uncertainty of the sensors and the environmental stability, the uncertainties of temperature, air pressure, and humidity are 35.2 mK, 10.3 Pa, and 2.1%, respectively, corresponding to 3.2 × 10^−8^. 2.7 × 10^−8^, and 2.3 × 10^−8^ air refractive index. The combined uncertainty related to the air refractive index is thus 5.2 × 10^−8^·*D*, i.e., 10.4 pm with 200 μm depth, which can be neglected. The second term in Equation (3) is related to the time delay *τ*. We use standard deviation to evaluation this part. From Figure 5 we find that the standard deviation can be below 3 μm when the output power is larger than 120 mW. Therefore, the second term can be calculated to 4.2 μm, which can be attributed to the program of the data processing and the output power of the laser, etc. To obtain better mechanical stability, we developed the small-size packages for the light source and the Michelson interferometer. In our system, the repetition frequency can also be precisely locked to a frequency reference, but the carrier-envelope-offset frequency is free. Based on the previous reports [25], the free-running carrier-envelope-offset frequency could result in about 150 μm uncertainty in the distance measurement. This problem can be relaxed because our system utilizes an equal-arm Michelson interferometer, and the carrier-envelope phase is the same for the measurement and the reference pulses. Finally, the combined uncertainty can be evaluated to 4.2 μm, showing good agreement with the measurement results (i.e., 4 μm). In the case of the screw, the standard deviation can be less than 5 μm when the irradiation power is larger than 120 mW, as shown in Figure 5. Consequently, the second term in Equation (3) can be calculated to 7.1 μm. The combined uncertainty can be thus evaluated to 7.1 μm for the screw measurement. Considering the spatial resolution, the *d*-axis resolution is related to the resolution of the distance measurement based on spectral interferometry. The lateral resolution is determined by the spot size (less than 1 μm in our cases) and the resolution of the two-axis platform (1 μm).

## 4. Conclusions

In conclusion, we describe a three-dimensional imager using frequency-comb spectral interferometry. We developed a compact, vibration-insensitive, and all-fiber frequency comb source where the mode-locked state is stimulated with the aid of a saturated absorber mirror. This type of all-fiber structure can well satisfy the severe environments of the real industry applications. Three-dimensional imaging has been playing significant roles in both science and technology. Based on the homemade laser source, we developed a three-dimensional imager based on the frequency-comb spectral interferometry. Our experimental results show a difference within 4 μm compared with the reference values. We also measured a screw specimen to describe the ability measuring a real workpiece with 7 μm measurement uncertainty. This level of accuracy and precision can satisfy most of the industry applications. To the best of our knowledge, frequency combs have not been used in real industry occasions, and further, not produced the economic benefit. Our work provides a powerful and accessible pathway to the future equipment using frequency combs, which can be used in the precision manufacturing of e.g., rockets, ships, and satellites. See Appendix A for a demonstration of the stabilized repetition frequency.

## Figures and Tables

**Figure A1:**
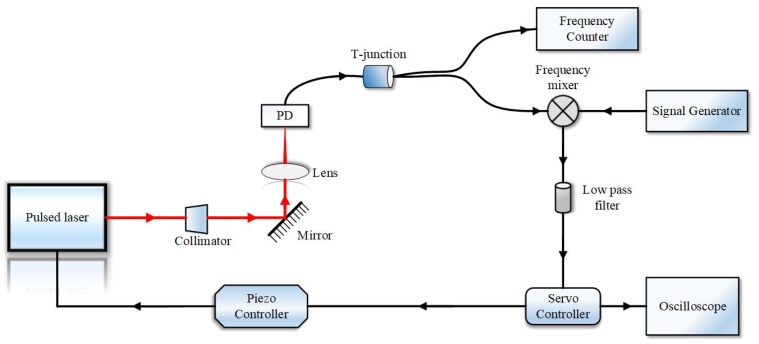
Locking scheme of the repetition frequency. PD: photodetector.

**Figure A2:**
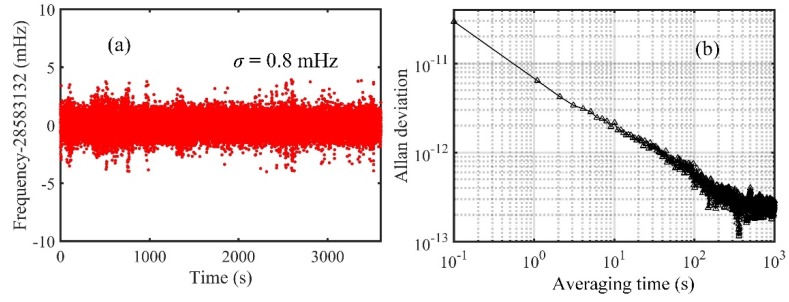
(**a**) Long-term measurement of the repetition frequency; (**b**) Allan deviation of the repetition frequency.

## Appendix A. Stabilization of the Repetition Frequency

The repetition frequency of the mode-locked laser can be locked simply by a phase locking loop, as shown in Figure A1. The mode-locked laser is detected by a fast photodetector (Thorlabs APD430C/M), whose output is composed of the fundamental and higher harmonic of the repetition frequency. The frequency counter (Agilent 53230A) is used to monitor the repetition frequency. The fundamental of the repetition frequency is mixed with the signal generator (Rigol DSG815) at the frequency mixer (Mini Circuits ZAD-2+), and the signal after the low pass filter (Mini Circuits BLP-1.9+) is the error signal, which is the input of the servo controller (New Focus LB1005). The output of the servo controller is amplified by a piezo controller (Thorlabs MDT694A), which can drive the intracavity piezo transducer to lock the repetition frequency.

Figure A2 shows the measurement results of the locked repetition frequency. The standard deviation is 0.8 mHz with 100 ms gate time. The Allan deviation is 6.4 × 10^−12^ at 1 s, and can further reduce to 2.8 × 10^−13^ at 1000 s, showing that the repetition frequency has been well locked to the frequency reference.
